# Deacetylation of ACO2 Is Essential for Inhibiting *Bombyx mori* Nucleopolyhedrovirus Propagation

**DOI:** 10.3390/v15102084

**Published:** 2023-10-12

**Authors:** Miao Hu, Yi You, Yao Li, Shiyi Ma, Jiaqi Li, Meng Miao, Yanping Quan, Wei Yu

**Affiliations:** 1 Institute of Biochemistry, College of Life Sciences and Medicine, Zhejiang Sci-Tech University, Hangzhou 310018, China; 2 Zhejiang Provincial Key Laboratory of Silkworm Bioreactor and Biomedicine, Hangzhou 310018, China

**Keywords:** *Bombyx mori*, BmNPV, ACO2, deacetylation, ATP

## Abstract

*Bombyx mori* nucleopolyhedrovirus (BmNPV) is a specific pathogen of *Bombyx mori* that can significantly impede agricultural development. Accumulating evidence indicates that the viral proliferation in the host requires an ample supply of energy. However, the correlative reports of baculovirus are deficient, especially on the acetylation modification of tricarboxylic acid cycle (TCA cycle) metabolic enzymes. Our recent quantitative analysis of protein acetylome revealed that mitochondrial aconitase (ACO2) could be modified by (de)acetylation at lysine 56 (K56) during the BmNPV infection; however, the underlying mechanism is yet unknown. In order to understand this regulatory mechanism, the modification site K56 was mutated to arginine (Lys56Arg; K56R) to mimic deacetylated lysine. The results showed that mimic deacetylated mitochondrial ACO2 restricted enzymatic activity. Although the ATP production was enhanced after viral infection, K56 deacetylation of ACO2 suppressed BmN cellular ATP levels and mitochondrial membrane potential by affecting citrate synthase and isocitrate dehydrogenase activities compared with wild-type ACO2. Furthermore, the deacetylation of exogenous ACO2 lowered BmNPV replication and generation of progeny viruses. In summary, our study on ACO2 revealed the potential mechanism underlying WT ACO2 promotes the proliferation of BmNPV and K56 deacetylation of ACO2 eliminates this promotional effect, which might provide novel insights for developing antiviral strategies.

## 1. Introduction

*Bombyx mori* is one of the reared economic insects worldwide. The silk and pupae of *Bombyx mori* have been used for silk weaving and pharmaceutical production for thousands of years [1]. As a model insect for genetics and immunology research [2], the insect is susceptible to *Bombyx mori* nucleopolyhedrovirus (BmNPV), which causes severe economic losses in sericulture [3]. BmNPV is a baculovirus that specifically infects silkworms and has a large circular double-stranded DNA genome with 143 putative open reading frames [4]. Silkworms and BmNPV constitute a well-established model of insect–virus interactions, including host immune response and immune escape from a virus. These findings provide a novel insight into viral infections and host defense [5]. In addition, the mechanism of energy metabolism pathways in viral infections and replications has always been a hot topic in virology research. Several physiological processes of viral infection require adequate energy, such as replication, transcription, and proliferation. However, due to a lack of cellular structures, viruses are unable to metabolize energy themselves and rely on the host’s cellular metabolic pathways to meet their energy demands [6]. Accumulating evidence suggested that viral infection induces the activation of multiple energy metabolic pathways in the host, including glycolysis and the tricarboxylic acid (TCA) cycle, which contribute to the rapid supply of energy and biomolecules for viral genome replication and virion assembly [7,8]. The TCA cycle is a universal energy metabolic pathway in eukaryotes. It is also the final metabolic pathway for glucose, protein, and lipid [9]. The study of energy metabolism in *Autographa californica* multiple nucleopolyhedroviruses (AcMNPV) showed that viral infection enhances the consumption of glucose in the host, which then enters the glycolytic pathway and TCA cycle [10]. In addition, AcMNPV infection drives a large amount of ATP synthesis by activating the expression of TCA cycle genes [11].

Mitochondrial aconitase (ACO2) is a prevalent metabolic enzyme in the TCA cycle, which reversibly catalyzes citric acid into isocitric acid through cis-aconitate acid [12]. As a protein bridging two key rate-limiting enzymes, citrate synthase (CS) and isocitrate dehydrogenase (IDH3), ACO2 activity is pivotal to maintaining ATP levels [12,13,14,15]. When ACO2 is knocked out in *Drosophila*, the TCA cycle will be impaired, resulting in decreased ATP levels [16]. In mitochondria, metabolic enzymes generally regulate their activity through acetylation modifications [17,18]. For example, ACO2 has 21 potential lysine (K) acetylation sites in mammals, and its enzymatic activity was markedly upregulated after acetylation [19]. Also, acetylation of ACO2 K567 increased the enzymatic activity, as observed in an enzyme kinetic study in *Escherichia coli* [20]. These findings indicated that acetylation modification may exert an essential regulatory role in ACO2 activity.

During the process of virus infection of host, the acetylation levels of many viral and host proteins change significantly [21,22,23]. The acetylation of proteins plays a vital role in virus–host interactions. These acetylated proteins exhibit two-sided properties, promoting viral replication or acting defensive mechanism for the host [24,25]. In BmN cells infected with BmNPV, we observed that ACO2 significantly attenuated the acetylation levels at K56 (0.72-fold) [21,22]. The reversible acetylation modification of metabolic enzymes in mitochondria is the central mechanism regulating protein activity [26,27]. Thus, changes in the acetylation level of ACO2 in *Bombyx mori* may exert functions by affecting energy metabolism during viral infection. Currently, due to the analogous positive electric charge, arginine (Arg, R) is taken to mimic K deacetylation. This method is used effectively in many studies to mimic the deacetylation of lysine [28,29]. Therefore, in this study, the K56R (Lys56Arg) of ACO2 was used to study the mechanism of deacetylation on the biological functions of metabolic enzymes and to further explore the regulatory mechanism of ACO2 deacetylation during BmNPV infection from the perspective of energy metabolism.

## 2. Materials and Methods

### 2.1. Cells, Viruses, and Vectors

The *Bombyx mori* ovarian cells (BmN cells) were preserved in our laboratory and cultured in Sf-900 (Gibco, Waltham, MA, USA) with 10% fetal bovine serum (Corning, Cambridge, MA, USA) at 27 °C. *Bombyx mori* nucleopolyhedrovirus (BmNPV) (Accession number: JQ991008) and BmNPV-EGFP [30] (enhanced green fluorescent protein) were propagated in our laboratory. Total mRNA was extracted from BmN cells by TRIzol reagent (Thermo Fisher Scientific, Watham, MA, USA) and reverse transcribed into cDNA (GenBank: XM_004932508.3) using RevertAid First Strand cDNA Synthesis Kit. pIEx-1 was an insert cell transient expression vector with His and Myc tags [30]. pIEx-1-ACO2 was synthesized by cloning *aco2* into a pIEx-1 vector using *Nde* I and *Pst* I restriction sites. Also, site-directed mutations in *aco2* (K56R) were synthesized by overlap polymerase chain reaction (PCR), as described previously [24]. All primers are listed in Table S1.

### 2.2. Antibodies

Aconitase antibody (1:2000 for Western blot) and baculovirus GP64 antibody (1:2000 for Western blot) were purchased from Santa Cruz Biotechnology, Dallas, TX, USA. Pan Ace-K antibody was obtained from PTM BIO, Hangzhou, China (1:1000 for Western blot, 1:50 for immunoprecipitation). Horseradish peroxidase (HRP)-conjugated His-tag and α-tubulin antibodies were procured from Proteintech, Rosemont, IL, USA; (1:10,000 for Western blot). Also, HRP-conjugated secondary antibodies were obtained from Biosharp Life Sciences Hefei, China (1:5000 for Western blot). Fugene 6 transfection reagent was bought from Promega Madison, WI, USA.

### 2.3. Transfection

Transfection was carried out by using Fugene6 transfection reagents (Promega) according to the manufacturer’s instructions. The transfection ratio was set as a plasmid (mass)/Fugene 6 (volume) = 1:3. BmN cells were seeded at a density of 1 × 10^6^ cells/well overnight. Then, each well was transfected with the plasmid and transfection reagent mixture.

### 2.4. Western Blot

Cells were lysed in 1% NP40, 150 mM NaCl, 1 mM ethylenediaminetetraacetic acid (EDTA), and 5 mM HEPES with a protease inhibitor cocktail (Thermo Fisher Scientific) on ice for 30 min. The protein concentration was estimated using Quick Start Bradford 1× Dye Reagent (Bio-Rad, Hercules, CA, USA). The cell extracts were separated by 10–12% sodium dodecyl sulfate-polyacrylamide gel electrophoresis (SDS-PAGE) and transferred onto polyvinylidene difluoride (PVDF) membranes (Merck Millipore, Billerica, MA, USA). The membranes were blocked with 5% skim milk in Tris-buffered saline with 0.01% Tween-20 (TBST) at 4 °C overnight and probed with the corresponding primary and secondary antibodies. Subsequently, the proteins were detected using SignalFire ECL Reagent (Cell Signaling Technology, Danvers, MA, USA) by Tanon 5500 Hypersensitivity Chemiluminescence Analyzer (Tanon, Shanghai, China) and quantified using gray value analysis by Image J software [31] (Bethesda, MD, USA).

### 2.5. Immunoprecipitation (IP)

With ACO2 acetylation levels analysis, the Pan Ace-K antibodies were diluted with IP lysis buffer (Thermo Fisher Scientific) and mixed with protein A/G magnetic beads (Thermo Fisher Scientific) at 4 °C overnight. BmN cells were lysed with IP lysis buffer containing 1% protease inhibitor for 30 min, followed by the addition of antibodies-magnetic beads and incubation for 2 h at 25 °C. The immunocomplexes were washed and acetylated proteins were analyzed by Western blotting.

### 2.6. Enzymatic Activity Analysis

ACO2, CS, and IDH3 activities were measured on visible spectrophotometry (Solarbio, Beijing, China). The mitochondrial proteins were extracted by differential centrifugation. Briefly, cells were lysed and centrifuged at 4 °C, 600× *g* for 5 min. Then, the supernatant was collected and centrifuged at 4 °C, 11,000× *g* for 15 min. The precipitate produced was mitochondria, which was lysed to obtain mitochondrial protein. Subsequently, the protein concentration was estimated using a BCA protein assay kit (Beyotime, Shanghai, China). Various reaction systems were configured according to the manufacturer’s instructions, and the OD values were obtained at the corresponding wavelengths to measure the enzyme activities.

### 2.7. ATP Levels Assay

Intracellular ATP content ratios were detected using Cell Titer-Glo^®^ Luminescent Cell Viability Assay (Promega) following the manufacturer’s instructions. The concentration of intracellular ATP was directly proportional to the intensity of fluorescence which was detected by using SpectraMax L Microplate Reader (Molecular Devices, San Jose, CA, USA). The protein concentration of each sample was detected by BCA regents to quantify ATP levels.

### 2.8. JC-1 Mitochondrial Membrane Potential (MMP) Assay

JC-1 is a mitochondrial fluorescent probe that can detect the MMP level [32]. When the MMP level is high, JC-1 aggregates in the matrix of mitochondria, forming polymers (J-aggregates), which can produce red fluorescence. In the lower MMP, JC-1 is a monomer, which can produce green fluorescence. Therefore, MMP was assessed according to the protocol of the JC-1 Mitochondrial Membrane Potential Assay Kit (Beyotime). Briefly, BmN cells were removed from the cell culture medium after being transfected for 72 h and washed with PBS twice. Then, cells were incubated with a JC-1 fluorescent probe at 27 °C for 30 min and washed three times with the JC-1 staining buffer. MMP was detected using a confocal laser microscope (IX81-FV1000, Olympus, Tokyo, Japan) at 488 nm (FITC) and 559 nm (Cy3) as well as keeping the same PTM voltage to control the background levels of emission. In addition, 10,000 BmN cells were analyzed by flow cytometry (NovoCyte Advanteon V6B5R3, Agilent, Santa Clara, CA, USA) under the same wavelength.

### 2.9. Fluorescence Microscopy Analysis of Virus Proliferation

The BmN cells were infected with BmNPV-EGFP for 48 h. The proliferation levels of BmNPV were observed under an inverted fluorescence microscope (Eclipse, TE2000-U, Nikon, Tokyo, Japan).

### 2.10. Determination of the Viral Titer

The cells were transfected with empty vector pIEx-1, pIEx-1-ACO2, and pIEx-1-ACO2-K56R, respectively, and then infected with BmNPV at a multiplicity of infection of 10 for 72 h. Subsequently, the virus in supernatant was harvested and serially diluted 10-fold from 10^−1^ to 10^−8^. A total of 100 μL of different gradient virus was inoculated into 96-well plates, and the data of well with 50% cells infected (cytopathic effect, CPE) were recorded at 0, 24, 48, 72, and 96 h p.i. The viral titer was calculated by TCID_50_ endpoint dilution assay.

### 2.11. Real-Time Fluorescence Quantitative PCR (qPCR)

Total RNA was extracted from BmN cells as described above. Then, the HiScript II 1st Strand cDNA Synthesis Kit (Vazyme, Nanjing, China) was used to obtain the cDNA and removed the gDNA according to the manufacturer’s instructions. The *Rp49* gene was used as an internal control [33,34,35]. The qPCR was carried out using a GoTaq qPCR Master Mix Kit (Promega) on an ABI Prism 7500 Sequence Detection System (Applied Biosystems, Foster City, CA, USA). The primers are listed in Table S1.

### 2.12. Statistical Analysis

All experiments were independently repeated at least three times, and statistical analyses were performed using GraphPad Prism 9 software (Version 9.0.0, La Jolla, CA, USA). Data were presented as mean ± standard deviation (SD). *t*-test and two-way analysis of variance (ANOVA) for multiple comparisons were used to compare the two groups.

## 3. Results

### 3.1. BmNPV Induces TCA Cycle Gene Expression to Activate Metabolism in BmN Cells

**Figure 1:**
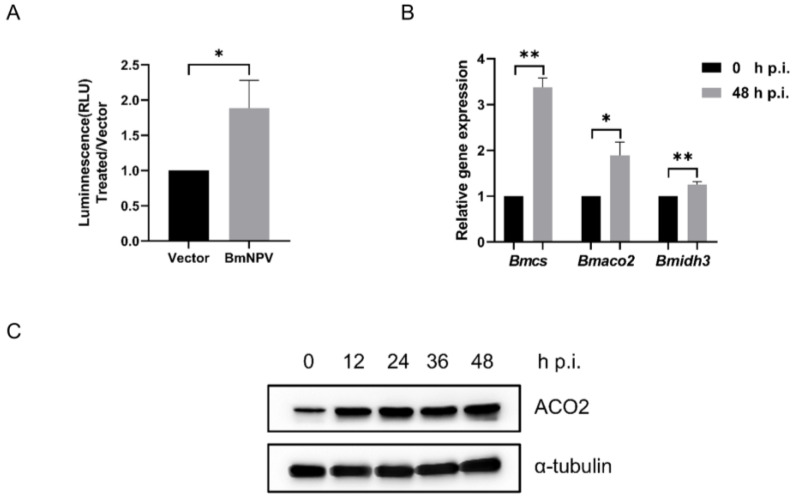
BmNPV infection promotes the TCA cycle. (**A**) BmNPV infected BmN cells. ATP levels were detected at 48 h post-infection (h p.i.). (**B**) Transcription levels of *Bombyx mori* TCA cycle genes, *Bmcs*, *Bmaco2*, and *Bmidh3*, were analyzed at 48 h p.i. *Rp49* was internal reference gene. (**C**) The protein levels of ACO2 were analyzed by anti-ACO2, and α-tubulin served as the loading control. “*” means *p* < 0.05, “**” indicates *p* < 0.01.

The proliferation of virus-infected host cells, including the processes of replication, transcription, and translation and assembly, requires a large supply of energy [36,37]. In order to study the effect of BmNPV on host energy metabolism after the invasion, intracellular ATP production was investigated. The results indicated that BmNPV infection significantly increases the cell ATP levels (Figure 1A). Then, the TCA cycle enzymes, closely related to ATP synthesis, were investigated by qPCR. The transcription analysis of *cs*, *aco2*, and *idh3* showed that the mRNA levels were upregulated after BmNPV infection for 48 h (Figure 1B). Due to the fact that ACO2 is the key metabolic enzyme bridging CS and IDH3 in the TCA cycle, we focused on the variations of ACO2 expression at different time points during BmNPV infection. We found that the expression of ACO2 increased after viral infection and was maintained until 48 h post-infection (Figure 1C). These results implied that BmNPV infection significantly increases the overall metabolic activation in BmN cells via the TCA pathway, and metabolic enzymes play a critical role after viral infection.

### 3.2. Deacetylation of ACO2 K56 Responds to BmNPV Infection in BmN Cells

**Figure 2:**
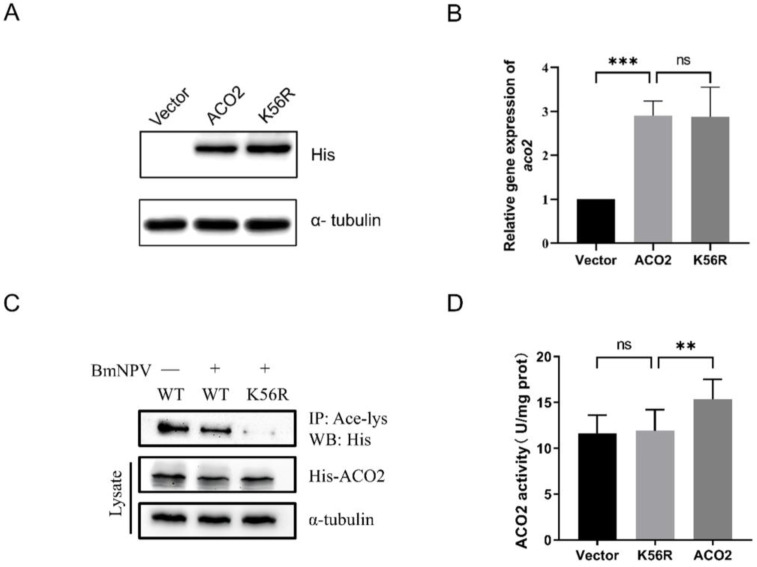
Effect of BmNPV infection on ACO2 deacetylation level and activity. (**A**) Detection of the protein expression level of WT-ACO2 and ACO2-K56R by Western blot; α-tubulin served as the control protein. (**B**) Detection of transcription level of *aco2* by qPCR post-transfection with pIEx-1-ACO2 and pIEx-1-ACO2-K56R for 72 h. *Rp49* was internal reference gene, and pIEx-1 was the control. (**C**) Post-transfection of WT and K56R ACO2 for 72 h, following BmNPV infection for 48 h. IP was performed with pan Ace-Lys antibody, and an anti-His tag was used to detect exogenous ACO2 and acetylated ACO2; α-tubulin served as the loading control. (**D**) Post-transfection with pIEx-1, pIEx-1-ACO2, and pIEx-1-ACO2-K56R for 72 h. The cellular ACO2 activity was analyzed using an ACO2 enzyme activity assay kit. “**” means *p* < 0.01, “***” means *p* < 0.001. “ns” means no significant difference.

Our previous acetylome analysis suggested that one potential acetylation site, K56 of *Bombyx mori* ACO2, undergoes deacetylation modification post-BmNPV infection [22]. In order to verify the variation of ACO2 acetylation level and acetylation position after BmNPV infection, the recombinant transient expression vector pIEx-1-ACO2 was constructed. The results showed that ACO2 overexpression was significant at 72 h post-transfection in BmN cells (Figure S1). This time point was suitable for subsequent experiments, wherein the effects of ACO2 overexpression and deacetylation were studied during the viral challenge. The overlap PCR was performed for site-directed mutagenesis, substituting lysine 56 with arginine (Lys56Arg; K56R) to mimic deacetylated lysine. Then, the BmN cells were transfected with recombinant wild-type (WT) ACO2 and deacetylation-mimic ACO2-K56R (Figure 2A,B). The results indicated that deacetylation-mimic ACO2-K56R and WT-ACO2 expression were similar, and the protein stability was unaffected. In the previous post-baculovirus challenge, only one lysine acetylation site (K56 residue) was determined and analyzed by HPLC/MS/MS (Figure S2). Therefore, IP of total acetylated-lysine proteins (pan Ace-K antibody) followed by immunoblotting with His-tag antibody confirmed the reduced acetylation levels of ACO2 after BmNPV infection (Figure 2C). Compared to WT ACO2, ACO2-K56R showed markedly reduced acetylation, further confirming that the expression of ACO2-K56R abolishes the acetylation marks on ACO2 and corroborates that ACO2 is acetylated at the K56 site.

Previous studies have shown that the acetylation level of mammalian ACO2 was closely related to the enzymatic activity and that the acetylation at the lysine site significantly enhances this activity [19]. In addition, the acetylation of aconitase K56 increased the enzymatic activity, as assessed by enzyme kinetic studies in *E. coli* [20]. In order to investigate the effect of *Bombyx mori* ACO2 deacetylation, we overexpressed WT and ACO2-K56R and analyzed the enzyme activity using the aconitase activity assay kit (Figure S3). The results showed that the enzymatic activity of ACO2-K56R was significantly lower than that of WT (Figure 2D), indicating that the deacetylation of K56 down-regulates the exogenous ACO2 activity and plays a key role in the enzymatic function.

### 3.3. Deacetylation of ACO2 K56 Affects Mitochondrial Functions

**Figure 3:**
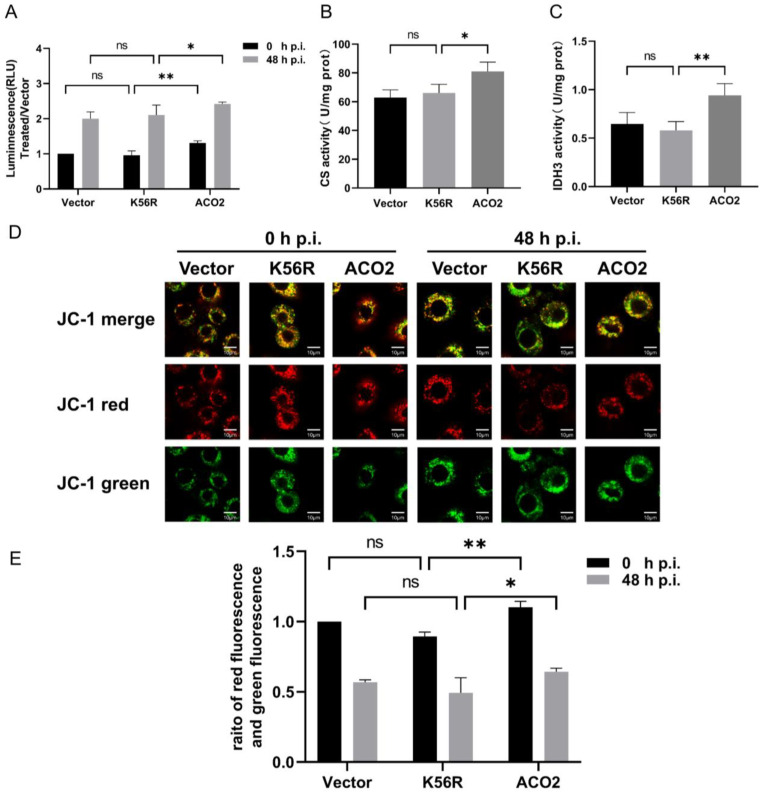
Effect of ACO2 deacetylation on mitochondrial energy metabolism. (**A**) BmN cells were transfected with pIEx-1, pIEx-1-ACO2, and pIEx-1-ACO2-K56R for 72 h. Intracellular ATP levels were analyzed by ATP assay kit after BmNPV infection for 48 h. ATP levels were proportional to fluorescence. (**B**,**C**) The activities of CS and IDH3 were analyzed using the enzyme activity assay kit. The enzyme activity was calculated based on the protein concentration. (**D**) MMP was observed under a confocal laser microscope after BmNPV infection for 48 h; scale bar: 10 μm. (**E**) MMP was analyzed by flow cytometry. “*” means *p* < 0.05, “**” means *p* < 0.01. “ns” means no significant difference.

ACO2 is one of the metabolic enzymes in the TCA cycle, and its altered activity affects cell energy metabolism [12]. The effect of ACO2 deacetylation on intracellular ATP level was assessed based on the above enzymatic activity results in BmN cells. The results showed that overexpression of WT ACO2 but not ACO2-K56R promotes intracellular ATP levels (Figure 3A). After BmNPV infection, the ATP level was the same as above (Figure 3A). Furthermore, we assessed the effect of the deacetylation of ACO2 on the activity of TCA cycle rate-limiting enzymes, including CS and IDH3. Compared to WT ACO2, ACO2-K56R decreased the activity of CS (Figure 3B) and IDH3 (Figure 3C) significantly. In order to evaluate the level of mitochondrial state, MMP was analyzed in BmN cells. The results of fluorescence (Figure 3D) and flow cytometry analysis (Figure 3E) showed that overexpression of WT ACO2 was more positive than ACO2-K56R for MMP. These data suggested that deacetylation of exogenous ACO2 K56 significantly affected the activities of CS and IDH3, decreasing the cellar energy metabolism and affecting MMP compared to WT exogenous ACO2.

### 3.4. Deacetylation of ACO2 K56 Inhibits the Proliferation of BmNPV

**Figure 4:**
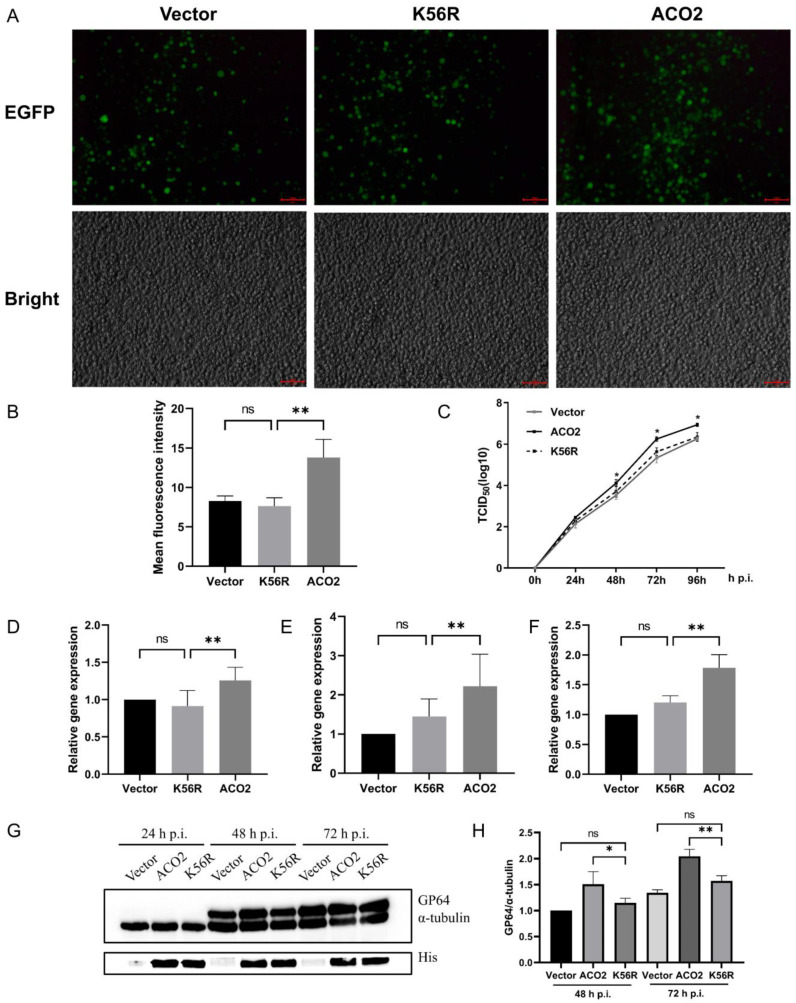
Deacetylation of ACO2 adverse to BmNPV proliferation. (**A**) BmN cells were transfected for 72 h following viral infection. ACO2 deacetylation on viral proliferation was analyzed by viral fluorescence intensity at 48 h p.i. with BmNPV-eGFP, and the bright field displayed cell number and growth states; the scale bar was 100 μm. (**B**) Fluorescence quantification of 4A was performed by Image J software, and the mean fluorescence intensity was analyzed. (**C**) After BmNPV infection for 96 h, viral supernatant was collected for TCID_50_ endpoint dilution and viral titer was analyzed. (**D**–**F**) Gene transcription levels of BmNPV *lef3* (**D**), *vp39* (**E**), and *gp41* (**F**) were analyzed. *Rp49* was internal reference gene. (**G**,**H**) BmNPV GP64 expression levels were analyzed by Western blot using anti-GP64 antibody; anti-His detected exogenous ACO2 level, and α-tubulin served as the loading control. “*” means *p* < 0.05, “**” means *p* < 0.01. “ns” means no significant difference.

Based on the above results, the *Bombyx mori* host reduces the acetylation level of ACO2 K56 in response to BmNPV infection. Next, we assessed the role of ACO2 deacetylation in BmNPV and the proliferative capacity of the virus by measuring the rate of proliferation based on BmNPV fluorescence and viral titer. Quantitative analysis of fluorescence intensity (Figure 4A,B) and viral titer (Figure 4C) indicated that the overexpression of WT ACO2 was beneficial to the proliferation of BmNPV, whereas ACO2-K56R inhibited the effect of WT ACO2 in promoting BmNPV replication, indicating that the deacetylation of ACO2 at the K56 residue was detrimental to viral proliferation. 

Interestingly, viral replication and assembly are closely related to energy metabolism. In order to explore the mechanism of ACO2 deacetylation inhibiting BmNPV replication, we selected BmNPV DNA replication, nucleocapsid, and virion assembly-related genes (*lef3*, *vp39*, and *gp41*) to analyze the effect of ACO2 deacetylation on the expression of these genes. The differences in the transcription levels indicated that WT ACO2 promoted the transcription of the above three genes, whereas ACO2-K56R did not enhance these genes’ expression (Figure 4D–F). These findings suggested that WT ACO2 promotes the expression of viral key genes related to viral DNA replication and early gene transcription rather than K56 deacetylated-ACO2. Then, the expression of baculovirus essential structural envelope protein (GP64) was investigated. Exogenous ACO2 facilitated the expression of BmNPV GP64 but the exogenous ACO2-K56R results were close to the vector control (Figure 4G,H). In conclusion, the exogenous WT ACO2 promoted the proliferation of the virus by affecting the energy metabolism of host cells and up-regulating the expression of BmNPV DNA replication, assembly-related genes, and the viral structural protein GP64, whereas K56 deacetylation modification of exogenous ACO2 eliminated the above ACO2 facilitating effects.

## 4. Discussion

BmNPV is a specific *Bombyx mori* pathogen that impedes agricultural development. However, the mechanism of interaction between BmNPV and *Bombyx mori* regarding energy metabolism is yet unclear. Therefore, we aimed to elucidate the molecular mechanism of the TCA cycle’s metabolic enzymes during BmNPV infection and the mechanism of ACO2 deacetylation in regulating BmNPV proliferation.

Viral proliferation in host cells requires significant energy supplements, including replication, transcription, and proliferation processes [37]. Therefore, most viruses upregulate energy metabolism by activating energy metabolic pathways such as the TCA cycle [7]. Herein, we found that energy metabolism was also enhanced in BmN cells during BmNPV infection, reflected by a significant increase in intracellular ATP levels. As the center of intermediates and energy metabolism, the TCA cycle occupies a critical position in various metabolic pathways [38]. A previous study showed that AcMNPV infection upregulated the expression of metabolic enzyme genes (*cs*, *aco2,* and *idh3*) in the host TCA cycle [10]. Therefore, we examined the transcription levels of these three genes in BmN cells after BmNPV infection. Our results showed that BmNPV was consistent with AcMNPV and upregulated TCA cycle genes’ transcript levels in the host. Mitochondrial ACO2 belongs to the family of iron-sulfur-containing dehydratases. It regulates oxygen-dependent ATP biosynthesis and is promoted in the expression and activity after BmNPV infection. Thus, ACO2 plays an essential role in BmNPV proliferation.

Interestingly, our previous acetylome study found that the acetylation levels of ACO2 K56 position in BmN cells significantly decreased after BmNPV infection [22]. In the present study, we substituted arginine for lysine to mimic deacetylation (K/R). IP showed that ACO2 underwent deacetylation modification at the K56 position after BmNPV infection. As a conserved class of post-translational modifications, acetylation of proteins regulates mitochondrial metabolic activities. Reportedly, ACO2 regulates its activity and function through acetylation modifications in mammals and *E. coli* [19,20]. Similarly, the deacetylation of exogenous ACO2 decreases the enzymatic activity in *Bombyx mori*, resulting in a significant depression of ATP levels in mitochondria compared to exogenous WT ACO2, indicating that the deacetylation of K56 may play a critical regulatory role in the biological function of ACO2.

CS and IDH3 are the key rate-limiting enzymes involved in the TCA cycle. Previous studies reported that the activities of CS and IDH3 were associated with ACO2 enzymatic activity [39,40,41]. The overexpression of ACO2 promoted the activity of CS and IDH3, whereas the ACO2 knockdown significantly reduced the activity [12,13,14]. We also found that overexpression of deacetylated ACO2 failed to enhance intracellular CS and IDH3 activities, indicating that cellular mitochondrial energy metabolism was suppressed by TCA cycle flux reduction via ACO2 deacetylation after BmNPV infection. Previous studies have shown that the activity of mitochondrial TCA cycle enzymes was correlated with mitochondrial membrane potential [42,43,44] and the level of MMP was positively correlated with the ATP level in *Bombyx mori* [45]. As expected, unlike exogenous WT ACO2 that enhanced the MMP, overexpression of deacetylated modified ACO2 in BmN cells was close to the vector control. The decrease in MMP is one of the early features of apoptosis [46], which is detrimental to the proliferation of the virus [47], suggesting that the host may down-regulate energy metabolism and MMP to counteract BmNPV proliferation through the deacetylation of ACO2. Therefore, we investigated whether the deacetylation of ACO2 influenced the proliferation of the virus and progeny virus production. Also, the expression of the crucial genes *lef3*, *gp41*, *gp64*, and *vp39* of BmNPV was less than exogenous WT ACO2. LEF3 is a single-stranded binding protein essential for baculovirus replication [48], and GP41, GP64, and VP39 play a vital role in the assembly and budding of viruses [49,50]. Our result implied that exogenous WT ACO2-induced energy enhancement promoted virion to replicate DNA and infected the surrounding cells. However, no promotional effect on viral proliferation was observed with overexpression of deacetylated modified ACO2.

In conclusion, BmNPV induces the expression of host TCA cycle genes to provide energy for its proliferation, in which ACO2 plays a key role. Exogenous ACO2 enhances the activity of intracellular ACO2 and upregulates energy metabolism and MMP, resulting in upregulation of the transcript levels of DNA replication and assembly-related genes in BmNPV, thus promoting the replication and proliferation of the virus. However, the host deacetylates K56 of ACO2 to reduce enzymatic activity and counteract the enhanced energy metabolism with respect to upregulated expression. Moreover, deacetylation of exogenous ACO2 reduces the energy enhancement and viral proliferation promotion by WT ACO2. As a product of interaction between *Bombyx mori* and BmNPV, deacetylated ACO2 plays a critical role in the host’s defense against the virus by affecting energy metabolism. Taken together, our results provide new insights into the molecular mechanism of ACO2 deacetylation in the process of baculovirus infection with respect to energy metabolism.

## Data Availability

All the data generated during the current study are included in the manuscript.

## Supplementary Materials

The following supporting information can be downloaded at: https://www.mdpi.com/article/10.3390/v15102084/s1, Figure S1: Expression of pIEx-1-ACO2 at 24 h, 48 h, and 72 h; Figure S2: Mass spectrometry analysis of ACO2 after BmNPV infection; Figure S3. Vector, His-ACO2 and His-ACO2-K56R were transfected to BmN cells, then the mitochondrial and cytosol compartments followed by immunoblotting with cytosolic and mitochondrial proteins α-tubulin and Hsp60 respectively. Table S1: The primers used in the study.
