# Supplementary material for: Deacetylation of ACO2 Is Essential for Inhibiting Bombyx mori Nucleopolyhedrovirus Propagation
Source: Viruses. 2023 Oct 12;15(10):2084. doi: 10.3390/v15102084 (PMC10612070; doi:10.3390/v15102084)
Supplement: Supplementary file 1 [file viruses-15-02084-s001.zip › SM/Figure S1.pdf]

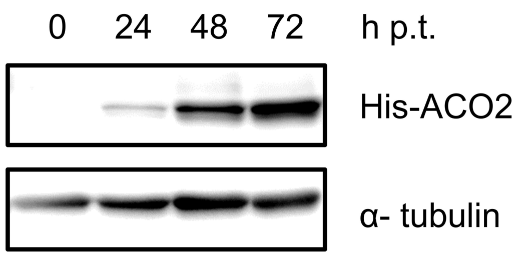

Figure S1. Expression of pIEx-1-ACO2 at 24 h, 48 h and 72 h, His-ACO2 detected the exogenous ACO2 expression and α-tubulin served as the loading control.
