# Supplementary material for: Deacetylation of ACO2 Is Essential for Inhibiting Bombyx mori Nucleopolyhedrovirus Propagation
Source: Viruses. 2023 Oct 12;15(10):2084. doi: 10.3390/v15102084 (PMC10612070; doi:10.3390/v15102084)
Supplement: Supplementary file 1 [file viruses-15-02084-s001.zip › SM/Figure S3.pdf]

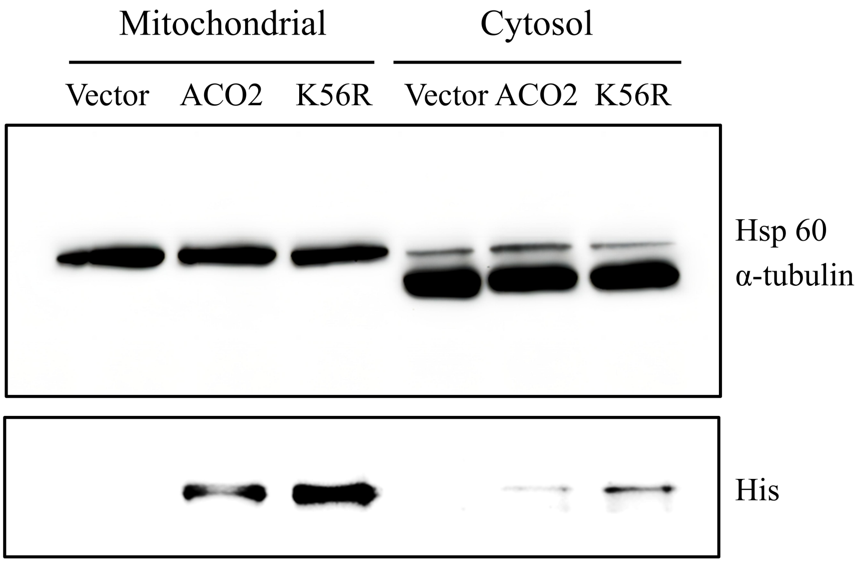

Figure S3. Vector, His-ACO2 and His-ACO2-K56R were transfected to BmN cells, then the mitochondrial and cytosol compartments followed by immunoblotting with cytosolic and mitochondrial proteins α-tubulin and Hsp60 respectively.
