# Supplementary material for: Deacetylation of ACO2 Is Essential for Inhibiting Bombyx mori Nucleopolyhedrovirus Propagation
Source: Viruses. 2023 Oct 12;15(10):2084. doi: 10.3390/v15102084 (PMC10612070; doi:10.3390/v15102084)
Supplement: Supplementary file 1 [file viruses-15-02084-s001.zip › SM/Table S1.pdf]

Table S1 The primers used in the study.

Name	Sequence
**pIEx-1-ACO2-F**	TTC**CATATG**ATGGCGCACTGTATGAGAGTTT
**pIEx-1-ACO2-R**	AA**CTGCAG**CTTTCCGGAAGCGATTTCCTT
**ACO2-K56R-F**	CGAAAAGTTGACGAGAAATTTAGAGGTGGT
**ACO2-K56R-R**	CCACCTCTAAATTTCTCGTCAACTTTTCG
***aco2*- F**	CTGCAACATGGGAGCTGAGA
***aco2*- R**	ATAGGGTGCCTTGTTGTCGG
***cs*- F**	CCCGAAGGTCTTTTCTGGCT
***cs*- R**	GACATGGGATGCAGTTTGCC
***idh3*- F**	TGCTTTTGGAGCCTTTTCGTG
***idh3*- F**	AGGGATGAGCGTAACCTTGC
***Rp49*-F**	TGCTCCCAAATGGATTCCGTAAG
***Rp49*-R**	CACGATCAGCTTCCGCTTCTTC
***lef3*- F**	CCGATTCGGATGACCGTTCT
***lef3*- R**	CGCCGTTTTCGACAGTTACG
***vp39*- F**	TTGACGAAACGGGTCTGGTG
***vp39*- R**	CGGAACGTACGTCGGGTATT
***gp41*- F**	CGTAGTGGTAGTAATCGCCGC
***gp41*- R**	AGTCGAGTCGCGTCGCTTT

The bold part is the restriction site, and the gray shaded part is the site-directed mutation.
