# Supplementary figures and images for: Deacetylation of ACO2 Is Essential for Inhibiting Bombyx mori Nucleopolyhedrovirus Propagation
Source: Viruses. 2023 Oct 12;15(10):2084. doi: 10.3390/v15102084 (PMC10612070; doi:10.3390/v15102084)

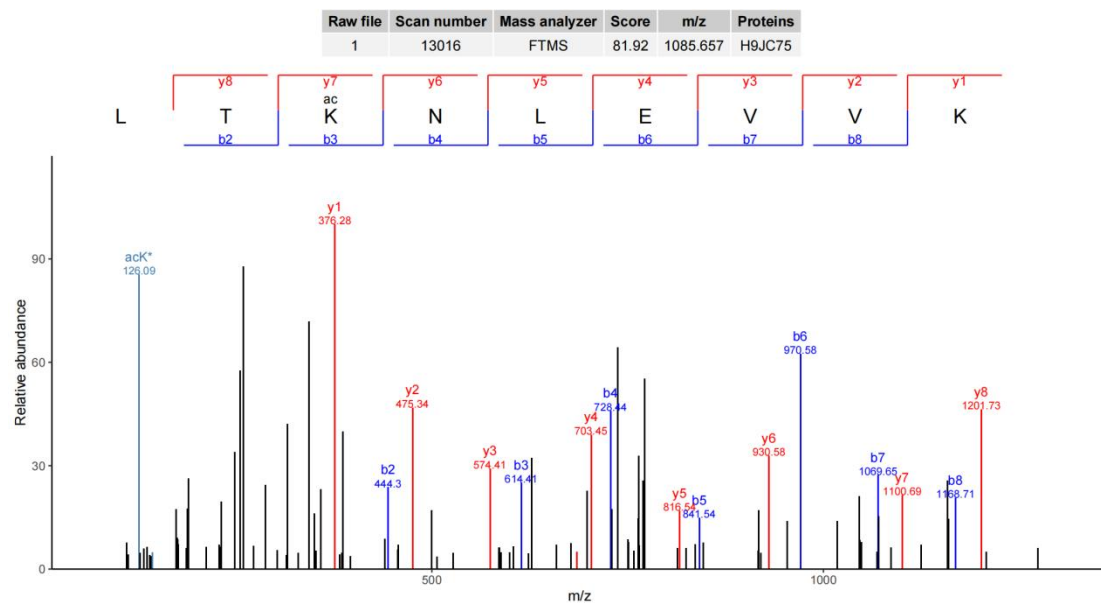

**Figure S2.** Mass spectrometry analysis of ACO2 after BmNPV infection.

Supplement: Supplementary file 1 [file viruses-15-02084-s001.zip › SM/Figure S2.pdf]
